# Artificial intelligence-based analysis of visual electrophysiological signals for clinical interpretation support

**DOI:** 10.3389/fnins.2026.1811969

**Published:** 2026-04-30

**Authors:** Mathieu Seraphim, Marie Alice Laville, Jean Claude Quintyn, Juliette Thariat

**Affiliations:** 1 Department of Radiation Oncology, Centre François Baclesse, Normandy University, Caen, France; 2 Department of Ophthalmology, University Hospital, Caen, France; 3 UNICAEN, ENSICAEN, CNRS/IN2P3, LPC Caen UMR 6534, Caen, France

**Keywords:** artificial intelligence, deep learning, detection, electroretinogram, interpretation, machine learning, visual evoked potentials

## Abstract

**Introduction:**

Visual electrophysiology, including electroretinograms (ERG) and visual evoked potentials (VEP), provides a real-time functional assessment of retinal and post-retinal pathways, complementing structural imaging. Subtypes such as transient, periodic, multifocal, and code-modulated signals probe distinct physiological mechanisms and reveal pathological signatures ranging from photoreceptor dysfunction to cortical pathway impairment. However, interpretation is often challenged by low signal amplitude, noise, and inter-individual variability. Advances in artificial intelligence (AI) enable automated, objective and reproducible analysis, and may improve sensitivity, and scalability in clinical and research environments. We undertook a literature review to identify the potential of automated analysis of brief visual electrophysiology signals to support medical interpretation in ophthalmology.

**Materials and methods:**

A review of the 2020–2025 literature was undertaken.

**Results:**

AI has been increasingly applied to ERG and VEP signals. These signals encode complex pathophysiological processes. Their features vary widely as they are transient (triggered by a single stimulus), periodic (repeated over time), multifocal (capturing signals from multiple visual field locations), or dependent on specific timing or coding schemes. These properties influence the choice of the most appropriate AI method for analysis. Classical ML methods remain useful for interpretable, feature-based classification of relatively scarce medical data, such as transient/aperiodic VEP and ERG. By modeling latent dynamics, AI can identify subtle or early dysfunction and harmonize interpretation across centers.

**Conclusion:**

AI supports reproducible, clinician-independent pipelines for electrophysiology, well-suited to high-volume clinics and large-scale screening. The convergence of standardized acquisition protocols with advanced AI analysis has the potential to deliver more personalized, timely, and objective assessments of visual system integrity in neuro-ophthalmic practice.

## Introduction

Electrophysiology has long been a cornerstone of functional assessment in ophthalmology and visual neuroscience, providing a direct window into the dynamic physiology of the visual system. In both routine and specialized clinical practice, techniques such as the electroretinogram (ERG) and the visual evoked potential (VEP) offer essential insights into retinal and post-retinal function (Mahroo, 2023; Robson et al., 2018). Unlike structural imaging modalities, electrophysiological recordings capture real-time electrical responses of visual tissues to controlled visual stimuli, enabling the detection of functional abnormalities that may precede irreversible structural damage and their patterns reflect functional topography across the visual system. Consequently, ERG and VEP play a complementary role alongside clinical examination and imaging in a wide range of conditions, including inherited retinal dystrophies; optic neuropathies, both inherited and acquired; demyelinating disorders; and radiation-induced injury.

ERG and VEP both assess stimulus-evoked neural activity, at the level of the retina for ERG and along the visual pathways up to the visual cortex for VEP. Each modality encompasses multiple standardized subtypes that probe distinct components of the visual system with varying spatial and functional specificity, as defined by the International Society for Clinical Electrophysiology of Vision (ISCEV) (Robson et al., 2018). Full-field ERG evaluates global retinal function, pattern ERG (PERG) preferentially reflects retinal ganglion cell activity, and multifocal ERG (mfERG) provides localized retinal response mapping across the posterior pole. Similarly, flash VEPs capture gross cortical responses, whereas pattern-reversal VEPs and multifocal VEPs (mfVEP) enable more precise assessment of visual pathway integrity and retinotopic cortical organization. Together, these protocols allow exploration of diverse physiological processes ranging from phototransduction and synaptic transmission to cortical integration, and reveal distinct pathological signatures associated with ischemia, hereditary degeneration, demyelination, or radiation exposure.

Beyond diagnosis, ERG and VEP are particularly well suited for early detection of dysfunction and longitudinal monitoring of treatment effects. Because they directly reflect neural function, electrophysiological measures can detect subtle physiological changes that occur before structural alterations become visible on imaging. This makes them valuable for monitoring disease progression as well as response to interventions, including pharmacological treatments, gene or cell therapies, and neurorehabilitation strategies such as perceptual learning or neuromodulation. Changes in waveform morphology, latency, or spatiotemporal organization may provide objective markers of functional recovery, stabilization, or deterioration over time, supporting treatment adaptation and personalized follow-up.

Despite their clinical value, ERG and VEP recordings remain challenging to interpret due to their low signal amplitudes, susceptibility to noise, substantial inter-individual variability, and dependence on expert visual interpretation. Artificial intelligence approaches, including machine learning (ML) and deep learning (DL)-based analysis of ERG and VEP signals holds promise for improving sensitivity to early functional impairment, disentangling overlapping signal components, and detecting subtle changes induced by treatment. DL approaches address several of these limitations by enabling the analysis of complete, high-dimensional ERG and VEP waveforms rather than relying exclusively on predefined readout parameters, as in classical analysis pipelines. By operating directly on raw or minimally processed time-series data, DL models can automatically learn informative signal representations. Electrophysiological time series exhibit complex and hierarchical temporal structures that are difficult to capture through manual feature engineering. As a result, DL-based methods can uncover subtle temporal relationships and waveform characteristics that may be overlooked when analysis is restricted to conventional descriptors such as peak amplitudes and implicit times. In clinical contexts, artificial intelligence approaches may reduce inter-reader variability, shorten interpretation time, and enable standardized, objective monitoring across repeated examinations. Importantly, these capabilities are particularly relevant for post-treatment follow-up, where small functional changes can inform therapeutic efficacy, guide rehabilitation strategies, and support timely intervention adjustments. Similar DL-driven frameworks have already demonstrated clinical utility in brain-computer interface applications (Edelman et al., 2025), where meaningful information is inferred directly from evoked neural responses.

In this review, we synthesize current artificial intelligence approaches best suited to the analysis of ERG and VEP subtypes, with the goal of advancing their use for detection, monitoring and automated analysis in ophthalmology.

## Methods

ERG and VEP signals present structured spatiotemporal patterns. AI models can be trained to uncover previously unrecognized signal phenotypes, recognize known pathological signatures in these signals, detect regional dysfunction, quantify progression, and even predict therapeutic response. In supervised learning, the model is trained using data that come with known outcomes, such as categories (for classification), numerical values (for regression), or specific regions within the input (for segmentation). In unsupervised learning, the data has no labels; the model tries to find hidden patterns or structures on its own, such as grouping similar data points. Classical machine learning approaches, such as support vector machines, random forests, k-nearest neighbors, and gradient boosting, typically rely on handcrafted features. These features may include peak amplitudes and latencies, inter-peak intervals, amplitude ratios, spectral power components (Fourier or wavelet transforms), entropy measures, and nonlinear descriptors (e.g., fractal dimension or Hjorth parameters). Feature selection and dimensionality reduction techniques such as principal component analysis or linear discriminant analysis are often applied to improve generalization and reduce overfitting. Deep learning models can learn hierarchical feature representations directly from raw or minimally processed waveforms. Convolutional neural networks (CNN) are effective for extracting local waveform morphology and spatial correlations, whereas recurrent architectures and temporal convolutional networks capture sequential dependencies and long-range temporal dynamics. Hybrid models can integrate spatial and temporal features to model complex electrophysiological behavior.

A systematic literature search was conducted in PubMed to identify studies focusing on ERG, VEP, and steady-state VEP [SSVEP] in conjunction with artificial intelligence (AI) methodologies (including machine learning, deep learning, convolutional neural networks, transformers, random forests, and neural networks). Search strategy combined terms for visual electrophysiology and AI in [Title/Abstract]: ((“electroretinogram” OR “electroretinography” OR “visual evoked potential” OR “SSVEP”) AND (“machine learning” OR “deep learning” OR “convolutional neural network” OR “transformer” OR “random forest” OR “neural network”)). Given the rapid development of AI methods within the past few years, this search was restricted to articles published between 2020/01/01 and 2025/07/31. Two authors (MS, JT) independently reviewed screened titles and abstracts and whether full original articles were available, in English and studying ERG, VEP, or SSVEP electrophysiology analyzed using AI/ML methods. Analyses of related but distinct electrophysiological responses (e.g., P300 wave, motor imagery) were rejected, as were hybrid analyses (simultaneous analyses of included and rejected signals). Disagreements were resolved by consensus. Additional articles were retrieved, mostly if treating of oncology/ophthalmology. The flow diagram and checklists were reported per PRISMA 2020 standards.

## Results

**Figure 1:**
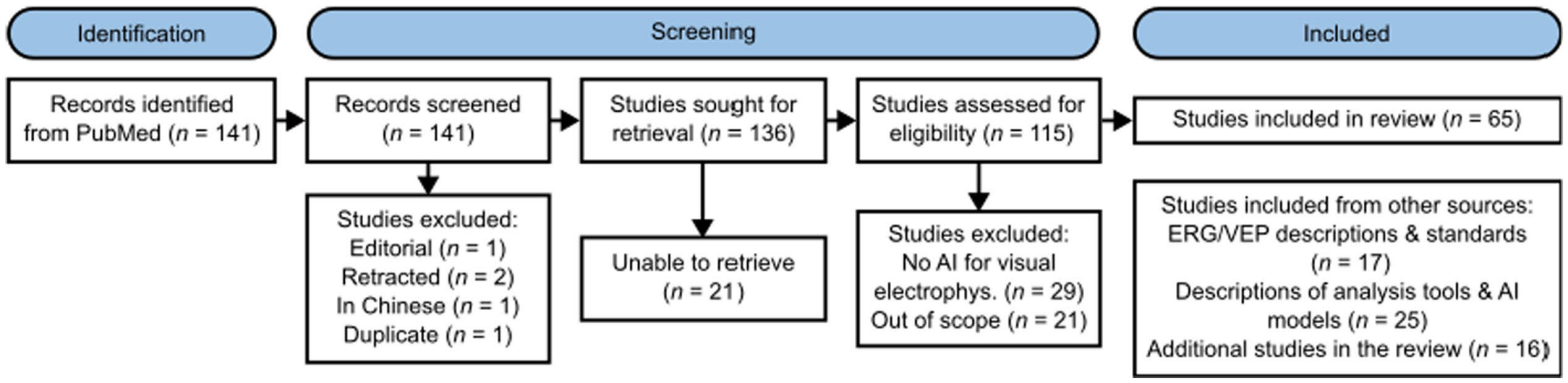
PRISMA diagram of the study.

A full breakdown of included studies is available in Figure 1.

### Data collection, cleaning and preprocessing in visual electrophysiology

All ML analyses require training data, with the quantity and quality of available data determining the scope of the analysis. Data scarcity can be a major limiting factor, as more complex models generally have higher training data requirements. Due to both medical regulatory concerns and the niche nature of visual electrophysiology, authors often have to contend with relatively small, single-center private datasets. As such, the field would benefit from increased collaboration between centers, to establish large, publicly available datasets with standardized acquisition protocols.

Even when using such standardized protocols, ERG and VEP recordings invariably capture noise, both physiological (non-visual brain activity, eye movements, muscle artifacts…) and non-physiological (electrical interference, electrode drift…). Consequently, they tend to exhibit a high signal-to-noise ratio. To lower this ratio, one can repeat the same recording multiple times, and averaging the resulting signals. While effective, this strategy is costly, and therefore most common with short, single-event stimulations. Setup-specific noise can also be captured through “dummy” recordings (e.g., replacing the patient with a saline bath), and thus be eliminated from subsequent recordings. When these strategies are not employed, or if some noise remains (e.g., the mains hum), further denoising and/or artifact rejection operations are usually applied. When entire frequency bands are deemed to be noise (e.g., frequencies below/above a threshold), they can be removed through filtering. When noisy signal components are more difficult to isolate, decomposition algorithms can be utilized to identify and delete them. Those include wavelet transforms (Ahmadi and Quian, 2013), empirical mode decomposition (Latifoglu et al., 2010) and independent component analysis (Zhang et al., 2024).

Manual noise assessment is prone to human biases. Instead, denoising algorithms can make use of machine learning. Adaptive filtering models, such as least mean squares, optimize the parameters of linear filters to match a desired output. More recently, CNN have been used to learn a series of convolutive digital filters to extract relevant information and discard unwanted data. As such, CNN designed as autoencoders (Kudaibergenova et al., 2022; Chuang et al., 2022) and generative adversarial networks (GAN) (Zeng et al., 2024) can denoise VEP signals. However, DL-based models often require large amounts of training data to be effective.

Artifact rejection is the detection and deletion of signal segments dominated by noise, such as eye movements or muscle activity. It is used to increase overall data quality, at the cost of quantity. Artifacts can be identified algorithmically, such as through thresholding (Zele et al., 2015) or through independent component analysis (Zhang et al., 2024). Machine learning models, like support vector machines (Abbaspour et al., 2019), can also be utilized for artifact detection. However, these methods remove information, run the risk of deleting relevant data and may not significantly compensate for the loss of relevant information within rejected segments, as shown in electroencephalograms similar to ERG and VEP (Delorme, 2023). Overall, increasing data quality at the cost of quantity can be counter-productive, especially if the available data is already scarce. Likewise, for DL analyses, these operations may be entirely unnecessary; as DL models are often capable of discarding noise regardless of preprocessing.

In addition to improving the quality of signals, some preprocessing pipelines also aim to increase its quantity, by creating new inputs from the training data. In image-based pipelines, this data augmentation is often done through direct transformations, such as flipping, cropping, resizing and/or rotating. Such transformations can be ill-advised for ERG and VEP signals, as amplitude and latency / frequency are often central to the analysis. Modifying theme therefore risks denaturing the signals and worsening performance (Wang et al., 2024). An alternative is to use self-supervised DL models, such as autoencoders and GAN (Kulyabin et al., 2024; Kulyabin et al., 2025; Pan et al., 2024; Wang et al., 2023; Komolovaitė et al., 2022) or spiking neural networks (Singanamalla and Lin, 2021), to learn the structure of the studied signals, using them to generate synthetic inputs to train the main ML model.

### Model validation

The training methodology of supervised and unsupervised machine learning models differ significantly. Unsupervised models are mainly used for data exploration, and can be trained using the entire available dataset. Supervised models are trained to learn a relationship between an input and an expected output. As such, they are prone to overfitting. A robust validation is therefore essential to ensure generalizability. Performance is assessed using metrics appropriate to the task (e.g., accuracy, sensitivity, specificity, area under the receiver operating curve (ROC) …). The choice of metric depends in part on the training goal (e.g., favoring high specificity), but it should also take the data distribution into account. Initial class imbalance commonly favors the predominant class, suggesting that balanced accuracy rather than overall average accuracy be used.

To detect overfitting, the dataset must be split into training, validation and test subsets, with the validation set used for hyperparameter optimization. In DL, it is standard practice to assess the model performance after each training cycle, stopping the training when it starts overfitting. When data is scarce, the dataset split can have a significant impact on the model’s performance. A solution to this instability is *k*-fold cross-validation (Scott et al., 2026), with each of the *k* folds defined by a different random split; such that the *k* test sets form a partition of the dataset. Then one model is trained per fold, with the final performance being the aggregation of all test set performances. Instability decreases as *k* increases, but the training time is also multiplied by *k*. To ensure that all subsets are unrelated, each patient’s signals must be segregated in a single subset (training, validation or test). Even then, the subsets will include signals compiled by the same team(s), using the same equipment. To avoid these biases, whenever possible, a so-called “external validation set” (i.e., a wholly independent dataset) should be used to further assess the model’s ability to generalize.

### Choice of the AI methods from ERG and VEP subtype and waveform or signal properties

The choice of the most appropriate AI method for analysis of ERG or VEP signals will depend on various properties. Data quality and quantity will restrict the range of applicable models. Poor quality data may require complex models to parse; while scarce data will be insufficient to properly train complex models. Imbalanced or non-representative datasets will likewise limit a model’s ability to generalize. Traditional or “classical” ML models (such as shallow neural networks, decision trees or support vector machines) are generally simple, and often require experts to manually reduce the amount of input data through feature engineering to avoid overwhelming the model. Commonly extracted features include peak amplitudes, latencies, inter-peak intervals, spectral power, entropy measures, or time-frequency coefficients. Unsupervised clustering of extracted features can be performed on these features. DL require larger amounts of data than classical ML and can be difficult to interpret. In turn, the nature of the studied signals will dictate a ML model’s input and expected output. Signals may be transient (triggered by a single stimulus), periodic (repeated over time), multifocal (capturing signals from multiple visual field locations), or dependent on specific timing or coding schemes.

ERG and VEP recordings used in clinical settings typically take the form of one or more 1-dimensional (1D) signals, each signal representing the voltage between two electrodes over time. To accurately capture the relevant parts of these signals, which often occur within the first 10–130 ms after a stimulus, recordings are typically sampled at a high rate, such as 1,000 times per second (1,000 Hz) or more, to capture rapid neural responses occurring within tens of milliseconds after stimulation. Some tests, such as those using transient (single-event) stimuli, produce responses that last between 100 and 400 ms. Other types of recordings, such as those using repeated or multifocal stimuli, may last several minutes to gather enough data across different conditions. The ERG and VEP subtypes are presented in Tables 1, 2, respectively.

**Table 1:** Sub-types of ERG exams.

Exam	Standard protocol	Signal characteristics	Use cases
Full-field (ffERG) (Robson et al., 2022).	Flash (≤5 ms). Monocular or binocular. Light- or dark-adapted eyes. Multiple flash strengths possible. Transient, or light-adapted periodic flashes, a.k.a. flicker ERG (30 Hz).	Single flash: typically, a negative peak (a-wave) followed by a taller positive peak (b-wave) at around 50 ms, with an underlying higher-frequency oscillatory potential. Periodic flashes: succession of shorter negative peaks and taller positive peaks. Shown signal lengths: 100 or 150 ms. Sampling frequency ≥1,000 Hz.	Distinguishing between outer and inner retinal dysfunctions, and between cone or rod system dysfunctions.
Pattern (PERG) (Thompson et al., 2024).	Reversal of checkerboard pattern. Preferably binocular. Transient. Standard check size: around 48’.	Succession of negative and positive peaks; most notably P50 (≈50 ms) and N95 (≈50 ms). Signal length ≤200 ms. Sampling frequency ≥1,000 Hz.	Assessment of cone system function within the macula, and of retinal ganglion cell function.
Steady-state pattern (SS-PERG) (Thompson et al., 2024).	As with PERG, but faster. Recommended: 16 reversals per second.	Main components: amplitude and phase shift. Signal length ≥6 cycles (375 ms). Sampling frequency ≥1,000 Hz.	Better evaluation of response amplitude and latency (through phase) than PERG.
Multifocal (mfERG) (Hoffmann et al., 2021).	Pattern of black or white scaled hexagons Preferably binocular Pseudo-random pattern evolution at 60–75 Hz, at least 4,095 steps.	Hexagonal grid of 1D signals. Typical waveform: succession of negative and positive peaks, termed N1, P1 and N2 (latencies < 100 ms). Full signal length: multiple minutes. Sampling frequency ≥ 1,000 Hz.	Assessment of function in discrete retinal regions.

Signal properties not explicitly mentioned in the reference (e.g., recommended signal length) were inferred from available figures and descriptions.

**Table 2:** Sub-types of VEP exams.

Exam	Standard protocol	Signal characteristics	Use cases
Flash (FVEP) (Odom et al., 2016; Šuštar Habjan et al., 2025).	Flash (≤5 ms) Monocular Transient.	Succession of negative and positive peaks; most notably N2 (≈90 ms) and P2 (≈120 ms). Shown signal length: 300 ms. Sampling frequency ≥1,000 Hz.	Assessment of prechiasmal function (asymmetries between eyes) and/or post-chiasmal function (asymmetries between occipital lobes). Variable response across typical subjects. Less reliable than pattern VEP; still offers complementary information.
Pattern onset/offset (Odom et al., 2016; Šuštar Habjan et al., 2025).	Switch between checkerboard and diffuse gray background. Monocular Transient Checkerboard and background should have the same average luminance. Standard check sizes: 15′ and 60’.	Succession of positive and negative peaks; most notably C1 (positive, ≈75 ms), C2 (negative, ≈125 ms) and C3 (positive, ≈150 ms). Shown signal length: 300 ms. Recommended onset signal length: 200 ms. Sampling frequency ≥1,000 Hz.	Assessment of prechiasmal function (asymmetries between eyes) and/or post-chiasmal function (asymmetries between occipital lobes). Detection of malingering. Effective in examination of patients with nystagmus.
Pattern reversal (PVEP) (Odom et al., 2016; Šuštar Habjan et al., 2025).	Reversal of checkerboard pattern. Monocular Transient Check sizes: variable.	Succession of negative and positive peaks; most notably N75 (≈75 ms), P100 (≈100 ms) and N135 / N145 (≈135/145 ms). Shown signal length: 300 ms. Max signal length: 500 ms. Sampling frequency ≥1,000 Hz	Assessment of prechiasmal function (asymmetries between eyes) and/or post-chiasmal function (asymmetries between occipital lobes). Preferred clinical VEP, less variable in waveform and timing than other VEP stimuli.
Steady-state (SSVEP), includes frequency-modulated (f-VEP) and phase-modulated (p-VEP) (Bin et al., 2009; Norcia et al., 2015).	Single or multiple periodic visual stimuli (flashes, patterns, images, etc.), either fixed or parametrically varying (sweep VEP). For multi-stimuli trials, each stimulus typically has a specific frequency.	Main components: amplitude and phase shift. Overlap between responses, leading to near-sinusoidal signals above 10 Hz. Presence of harmonics for non-sinusoidal stimuli and non-linear systems. Multi-stimuli: multiple stimulus frequencies may be present in the signal, with the strongest frequency typically indicative of a fixation target. Shown signal lengths: 1 s to multiple minutes. Sampling frequency unspecified; would depend on the frequency of stimuli (shown: up to 35 Hz).	Measurement of spatial acuity; assessment of the visual field, binocular rivalry, visual attention. Cognitive response to stimuli reflected in the recordings → uses in cognitive research and BCI systems.
Time-modulated (t-VEP) (Bin et al., 2009).	Multiple looping, non-overlapping sequences of flashes, spaced to elicit distinct FVEP responses. Cycle period: ≈1 min.	FVEP responses specific to the fixation target.	Visual command selection in BCI systems.
Code-modulated (c-VEP) (Bin et al., 2009; Martínez-Cagigal et al., 2021).	Multiple stimuli flickering in a loop according to the same pseudo-random binary sequence, with offsets between stimuli Cycle period: ≈1 min.	Consistent response to the binary sequence. After a calibration period, the fixation target can be determined by the corresponding offset in the signal. Sampling frequency unspecified; signals appear sampled at least 1,000 Hz.	Visual command selection in BCI systems.
Motion-onset (m-VEP) (Kuba et al., 2007).	Motion (radial, spiral, rotating, translating.) of a pattern. Duration: 100–200 ms.	Succession of negative and positive peaks; most notably P1 (≈130 ms), N2 (≈160–200 ms) and P2 (≈240 ms), with P1 or N2 dominant depending on pattern and motion. Shown signal length ≤450 ms. Sampling frequency unspecified; signals appear sampled at around 1,000 Hz.	Diagnosis of optic neuritis/multiple sclerosis, neuroborreliosis, glaucoma, amblyopia, dyslexia, congenital nyctalopia or encephalopathies.
Multifocal (mfVEP) (Hood et al., 2003; Balachandran et al., 2003).	Pattern of black or white scaled squares. Monocular or binocular. Pseudo-random pattern evolution, similar frequency/number of steps to mfERG.	2D grid of responses extracted from a single signal. Typical waveform: succession of negative and positive peaks; most notably C1 (negative, ≈65 ms) and C2 (positive, ≈95 ms). Full signal length: multiple minutes. Sampling frequency unspecified; periods of less than 10 ms mentioned.	Ruling out non-organic visual loss. Diagnosis of optic neuritis/multiple sclerosis.

Signal properties not explicitly mentioned in the reference (e.g. recommended signal length) were inferred from available figures and descriptions.

The wide variety of ERG and VEP subtypes explored in this review demonstrates how electrophysiological signals, despite their apparent simplicity, encode complex physiological and pathophysiological processes, and how their distinct features determine the choice of the AI methods in ophthalmology.

### Protocol-specific characteristics of ERG and VEP with implications for AI models

ERG and VEP models have been used to help classify or predict clinically relevant outcomes, based on labeled examples, such as signals known to come from healthy individuals or from patients with specific visual or non-visual disorders. Supervised models include base models like linear (Su et al., 2012) and logistic regression (Stoltzfus, 2011), which model relationships between signal features and outcomes. Other commonly used techniques include statistical classifiers such as linear and quadratic discriminant analysis (Ghojogh and Crowley, 2019), and basic probabilistic models such as naïve Bayes models (Webb, 2017). Other approaches include support vector machines, which find the best boundary between groups; distance-based methods like minimum distance to mean models (Congedo et al., 2019) and k-nearest-neighbors models (Taunk et al., 2019); and decision trees (Suthaharan, 2016), that make decisions by asking a series of binary questions. Some studies also used shallow (non-deep) neural networks, most often in the form of perceptrons (Popescu et al., 2009). These models can be combined in so-called ensemble models, such as random forests (Breiman, 2001) and boosting algorithms (Schapire, 2013). Unsupervised clustering algorithms are more rarely used, but at least one approach uses a hierarchical agglomerative clustering algorithm (Murtagh and Legendre, 2014). These models are particularly useful for identifying abnormal patterns or predicting clinical status based on signal properties.

ERG and VEP recordings can be directly analyzed by DL models, notably through CNN (O’shea and Nash, 2015), either using a preexisting architecture (Kiranyaz et al., 2021) or coding their own. Signals can also be represented as 2D images (see later), utilizing image-based CNN models (Anumol, 2023). The signals can also be analyzed through DL architectures designed for timeseries analysis, such as recurrent neural networks like the Long Short-Term Memory model (Staudemeyer and Morris, 2019). A more recent alternative can be found in Transformer models, which utilize a self-attention mechanism to identify the most relevant elements in a sequence (Vaswani et al., 2017). This led to variants like the timeseries Transformer (Zerveas et al., 2021) and visual Transformer (Dosovitskiy et al., 2020).

A recap of all models presented in this article can be found in Tables 3, 4.

**Table 3:** Classical machine learning models used to analyze clinical ERG and/or VEP signals.

Model	Use cases	Pros and cons
Canonical correlation analysis	Periodic signal decomposition (Xu et al., 2023a; Besharat et al., 2024; Yao et al., 2022), component frequency detection (Besharat et al., 2024; Yao et al., 2022; Chailloux Peguero et al., 2023; Wang et al., 2023; Ravi et al., 2020; Xu et al., 2023b; Chen et al., 2024; Ingel and Vicente, 2021; Chen et al., 2023; De la Cruz-Guevara et al., 2021; Chuang et al., 2022; Ikeda and Washizawa, 2021; Ding et al., 2021; Wan et al., 2023; Dai et al., 2025; Lei et al., 2024), c-VEP analysis (Martínez-Cagigal et al., 2021; Dong et al., 2025).	State-of-the-art performance; but limited to periodic signals
Linear regression	Transient signal analysis (Gaillet et al., 2021).	Wide variety of models to pull from, fast training, and explainable results; but usually require manual feature extraction, and are limited in modeling complexity.
Logistic regression	Transient signal analysis (Glinton et al., 2022; Peredo et al., 2022; Banijamali et al., 2024), periodic signal analysis (Scott et al., 2026), multifocal signal analysis (Karaman et al., 2025).	
Least absolute shrinkage and selection operator (LASSO)	Transient signal analysis (Manjur et al., 2022), periodic signal analysis (De la Cruz-Guevara et al., 2021)	
Linear discriminant analysis (LDA)	c-VEP analysis (Martínez-Cagigal et al., 2021).	
Naïve bayes	Periodic signal analysis (Lei et al., 2024), multifocal signal analysis (Banijamali et al., 2024; Gajendran et al., 2022; Karaman et al., 2025), c-VEP analysis (Martínez-Cagigal et al., 2021).	
Support vector machine	Artifact detection (Abbaspour et al., 2019), transient signal analysis (Glinton et al., 2022; Gaillet et al., 2021; Manjur et al., 2023; Posada-Quintero et al., 2023; Constable et al., 2024; Gajendran et al., 2022; Noguez Imm et al., 2023; Diao et al., 2021; Hao et al., 2025), periodic signal analysis (Chen and Golomb, 2023; Chen et al., 2021; Lei et al., 2024), multifocal signal analysis (Habib et al., 2022; Banijamali et al., 2024; Karaman et al., 2025), c-VEP analysis (Martínez-Cagigal et al., 2021).	
Decision tree (DT)/random forest/DT + boosting	Transient signal analysis (Glinton et al., 2022; Zhdanov et al., 2022; Manjur et al., 2023; Posada-Quintero et al., 2023; Constable et al., 2024; Gajendran et al., 2022; Noguez Imm et al., 2023; Diao et al., 2021; Deng et al., 2021; Hao et al., 2025), periodic signal analysis (Scott et al., 2026), multifocal signal analysis (Banijamali et al., 2024).	
K-nearest neighbors	Transient signal analysis (Manjur et al., 2023; Posada-Quintero et al., 2023; Constable et al., 2024; Gajendran et al., 2022; Diao et al., 2021), multifocal signal analysis (Banijamali et al., 2024).	
Riemannian MDM	Transient signal analysis (Schwitzer et al., 2022), component frequency detection (Chevallier et al., 2021).	Requires complex representation (manifold of symmetric positive definite matrices); but simple and effective within the manifold.
Shallow neural network	Transient signal analysis (Yapici et al., 2021; Posada-Quintero et al., 2023; Constable et al., 2024; Heinrich et al., 2021), multifocal signal analysis (Karaman et al., 2025).	Can take the full signal as input.
Clustering	Transient signal analysis (Peredo et al., 2020), multifocal signal analysis (del Ortiz Castillo et al., 2020).	Well suited to data exploration.

**Table 4:** Deep learning models used to analyze clinical ERG and/or VEP signals.

Model	Use cases	Pros and cons		
Autoencoder/Adversarial network	Noise reduction (Kudaibergenova et al., 2022; Zeng et al., 2024; Chuang et al., 2022), synthetic signal generation (Kulyabin et al., 2024; Kulyabin et al., 2025; Pan et al., 2024; Wang et al., 2023; Komolovaitė et al., 2022), feature extraction (Chen et al., 2021).	Can be self-supervised (same input as output).		
Spiking neural network	Synthetic signal generation (Singanamalla and Lin, 2021).	Mimics the structure of biological neural networks. Theoretically adapted to biological signal processing, but relatively niche.		
1D CNN classifier	Transient signal analysis (Liang et al., 2023; Kulyabin et al., 2024; Giap et al., 2024), periodic signal analysis (Xu et al., 2023a; Zhao et al., 2022; Wang et al., 2023; Yue et al., 2025; Albahri et al., 2023; Ravi et al., 2020; Xu et al., 2023b; Guney et al., 2022; Wang et al., 2023; Bhuvaneshwari et al., 2023; Chen et al., 2023; Li et al., 2024; Chuang et al., 2022; Ikeda and Washizawa, 2021; Mahmood et al., 2022; Ding et al., 2021; Wan et al., 2023; Lei et al., 2024), multifocal signal analysis (Banijamali et al., 2024), c-VEP analysis (Dong et al., 2025).	Automatic feature extraction, can model highly complex relationships; but require large amounts of training data.	Do not require data transformation.	
1D recurrent network	Transient signal analysis (Kulyabin et al., 2024; Cheker et al., 2022; Diao et al., 2021), periodic signal analysis (Xu et al., 2023a; Albahri et al., 2023; Liu et al., 2024; Wang et al., 2024).			Designed for timeseries analysis; but outclassed by transformers.
Timeseries transformer	Transient signal analysis (Kulyabin et al., 2024; Kulyabin et al., 2024; Kulyabin et al., 2025).			State-of-the-art; but relatively costly.
2D CNN classifier	Transient signal analysis (Kulyabin et al., 2025; Kulyabin et al., 2023; Albasu et al., 2024) periodic signal analysis (Xu et al., 2023a; Yao et al., 2022; Chen et al., 2024; Israsena and Pan-Ngum, 2022; Ding et al., 2024; Albahri et al., 2023; Gao et al., 2022; Deng et al., 2023; Komolovaitė et al., 2022; Wan et al., 2023; Dai et al., 2025; Huang and Wei, 2025; Wei et al., 2025), multifocal signal analysis (Klistorner et al., 2022).		Model weights pre-trained on large datasets publicly available; but require a 2D transformation.	
Visual transformer	Transient signal analysis (Kulyabin et al., 2025; Kulyabin et al., 2023; Albasu et al., 2024; Kulyabin et al., 2023).			State-of-the-art; but relatively costly.
Purpose-built transformer	Periodic signal analysis (Wang et al., 2023; Wan et al., 2023; Ding et al., 2024; Yue et al., 2025; Qin et al., 2024; Chen et al., 2023; Wan et al., 2023).			

### Analysis of transient visual responses

Transient ERG and VEP waveforms are triggered by a single stimulus, often either flash-based or pattern-based. In medical practice, they are mainly described by recognizable waveforms, especially the amplitude and latency (time since stimulation) of characteristic peaks.

Both pattern ERG (PERG) (Thompson et al., 2024) and pattern-reversal VEP (PVEP) (Odom et al., 2016) utilize the sudden reversal of a black-and-white checkerboard. The former, described by the positive peak around 50 ms (P50) and the negative peak around 95 ms (N95), measures retinal ganglion cell function. The latter is described by the N75, P100 and N135 peaks, and is used to assess the integrity of the central visual pathway and visual conduction abnormalities. Other VEP subtypes utilize a switch from a gray background to a checkerboard of same luminance, or vice-versa (pattern onset or offset). These signals are clinically important for detecting early dysfunction, even in the absence of visual acuity loss.

**Figure 2:**
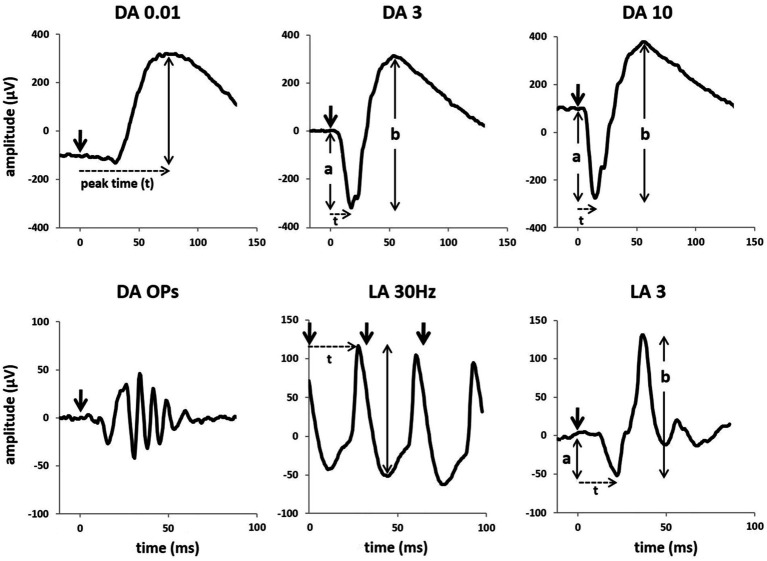
Full-field ERG responses. DA, dark-adapted; LA, light-adapted; OPs, oscillatory potentials; 0.01/3/10: flash intensity in phot cd·s·m^−2^; 30 Hz: flicker ERG stimulus frequency. Licensed under CC BY 4.0 (Robson et al., 2022), https://creativecommons.org/licenses/by/4.0/, unchanged.

Flash-based ERG, known as full-field ERG (ffERG), vary both by flash intensity and ambient lighting (Robson et al., 2022), with transient components characterized by their a-wave and b-wave. Dark-adapted ffERG, used to assess rod-driven retinal pathways, are usually triggered by a single flash of either low or high intensity (Figure 2). Light-adapted ffERG flashes typically have a fixed intensity. By contrast, flash-VEP (FVEP) (Odom et al., 2016; Šuštar Habjan et al., 2025) are fully transient, and less emphasis is put on flash intensity or lighting. FVEP detect gross visual pathway dysfunction, though they lack sensitivity and spatial precision, and exhibit high inter-individual variability and lower reproducibility than PVEP (Calcagni et al., 2024; Seok et al., 2018).

Motion-onset VEP (m-VEP) are non-ISCEV VEP variants, which utilize the change in motion of a pattern as stimulus, such as the expansion or contraction of a pattern of concentric rings (Kuba et al., 2007). m-VEP assess the function of the magnocellular visual pathway. They are used to evaluate motion perception and detect early visual deficits, particularly in conditions like glaucoma, optic nerve disorders, and demyelinating diseases, even when visual acuity and standard VEPs are normal.

Because the stimuli and resulting signals follow standardized timing and structure, transient ERG and VEP signals are well suited to ML approaches. They can be analyzed in the time domain, or through a time-frequency representation. DL-based time-domain analysis may also be done but signals should be raw or minimally altered. DL-based analysis would then rely on 1D CNN (Liang et al., 2023; Kulyabin et al., 2024; Giap et al., 2024), recurrent neural networks (Kulyabin et al., 2024; Cheker et al., 2022; Diao et al., 2021), or timeseries Transformers (Kulyabin et al., 2024; Kulyabin et al., 2024; Kulyabin et al., 2025). A frequency-based analysis of these signals could also be relevant, but as transient ERG and VEP signals are transient, e.g., aperiodic, a simple Fourier transform would have limited interest. Instead, time-frequency representations can be constructed, either through short-term Fourier transforms (STFT) or wavelet transforms (Kıymık et al., 2005). The resulting images can therefore be analyzed through 2D CNN or visual Transformer (Kulyabin et al., 2025; Kulyabin et al., 2023; Albasu et al., 2024; Kulyabin et al., 2023).

For classical ML, these inputs can be “condensed” through manual feature extraction. Notably, the amplitude and latency of characteristic peaks are often extracted to train supervised (Glinton et al., 2022; Peredo et al., 2022; Gajendran et al., 2022) or unsupervised (Peredo et al., 2020) ML models. Additional features are often extracted through statistics (mean, median, variance, kurtosis, entropy…), from the time domain (Gaillet et al., 2021; Banijamali et al., 2024; Gajendran et al., 2022; Noguez Imm et al., 2023; Diao et al., 2021; Deng et al., 2021; Heinrich et al., 2021; Hao et al., 2025), or from time-frequency representations (Yapici et al., 2021; Zhdanov et al., 2022; Schwitzer et al., 2022; Manjur et al., 2022; Gaillet et al., 2021; Gajendran et al., 2022). Other transformations, such as variable-frequency complex demodulation (Gasquet and Wootton, 1997), have been used for statistical feature extraction (Manjur et al., 2023; Posada-Quintero et al., 2023; Constable et al., 2024).

Finally, the choice of DL versus classical ML will in part depend on information density within signals. If the signals contain no relevant information beyond peak features, then a classical ML model should be sufficient. Inversely, analyzing information-poor signals (such as FVEP) with DL models might lead to overfitting. As such, DL models should only be considered if relevant information that cannot be captured with feature engineering is present outside of characteristic peaks.

### Analysis of periodic visual responses

Periodic ERG and VEP responses, usually triggered by periodic stimuli, are characterized primarily by amplitude and phase rather than discrete peaks. These include some ffERG responses (Robson et al., 2022). High-pass filtering (75 Hz) on higher intensity, dark-adapted, transient ffERG can reveal so-called oscillatory potentials. These are high-frequency wavelets reflecting inner retinal function, particularly amacrine cell activity, and sensitive indicators of early retinal vascular or ischemic dysfunction (Midena et al., 2021). The ISCEV also defines two types of periodic ERG stimuli. Flicker ERG are light-adapted ffERG using periodic flashes (Robson et al., 2022), reflecting the function of long- and medium-wavelength cones while receiving minimal contribution from short-wavelength cones. Steady-state PERG (SS-PERG) use periodic pattern reversals (Thompson et al., 2024), and offer a more objective evaluation of the response’s amplitude and latency (through phase) than the standard PERG.

Steady-state VEP (SSVEP) are non-ISCEV and primarily used in brain-computer interfaces (BCI) (Bin et al., 2009; Norcia et al., 2015). They utilize one or more periodic signals (usually flashes) as stimuli, and generate frequency-locked, quasi-sinusoidal responses with identifiable harmonics. SSVEP measure the brain’s continuous electrical response to visual stimuli flickering at constant frequencies, reflecting how well the visual system can synchronize with repetitive input.

Stimuli can be fixed, or change gradually over time, as in so-called ‘sweep VEP’ protocols. In particular, the frequency (frequency modulation, or f-VEP) and/or phase (phase modulation, or p-VEP) of the concentrated-upon stimulus is extracted, which can be used to send commands.

Most DL approaches applicable to transient (single stimulus) signals are generic enough to be applied to any 1D data, including periodic signals. DL models can extract relevant features from 1D temporal signals (Xu et al., 2023a; Zhao et al., 2022; Wang et al., 2023; Yue et al., 2025; Albahri et al., 2023; Ravi et al., 2020; Guney et al., 2022; Pan et al., 2024; Liu et al., 2024; Wang et al., 2023; Bhuvaneshwari et al., 2023; Chen et al., 2023; Chuang et al., 2022; Mahmood et al., 2022; Ding et al., 2021; Wan et al., 2023; Dai et al., 2025; Wang et al., 2024; Lei et al., 2024) but periodic signals are often best analyzed through their 1D Fourier transform; both directly through DL (Xu et al., 2023a; Xu et al., 2023b; Li et al., 2024; Ikeda and Washizawa, 2021; Ding et al., 2021; Dai et al., 2025) or through classical ML pipelines. Time-frequency representations (e.g., STFT, wavelet transforms…) seem less relevant, as the temporal dimension offers little information. This said, these representations are two-dimensional, allowing for the use of powerful image-based DL models (Xu et al., 2023a; Gao et al., 2022). Alternatively, SSVEP recordings can be formed into 2D inputs by stacking multiple channels and/or isolated frequency bands together, and thus compatible with image-based models (Yao et al., 2022; Chen et al., 2024; Wang et al., 2023; Wan et al., 2023; Israsena and Pan-Ngum, 2022; Ding et al., 2024; Yue et al., 2025; Qin et al., 2024; Deng et al., 2023; Komolovaitė et al., 2022; Wan et al., 2023; Dai et al., 2025; Huang and Wei, 2025; Wei et al., 2025).

Though a DL analysis of SSVEP may be effective, it is often not necessary; as a large number of classical approaches have been developed for, and successfully applied to, these signals. Canonical correlation analysis (Weenink, 2003) establishes a link between two multivariate variables, such as SSVEP channels on one hand and frequency harmonics on the other. From there, it can be used to identify the concentrated-upon stimulus in frequency-modulated SSVEP. Canonical correlation analysis and its variants (Besharat et al., 2024) are therefore often utilized to identify the concentrated-upon stimulus in frequency-modulated SSVEP (Besharat et al., 2024; Chailloux Peguero et al., 2023; Wang et al., 2023; Ravi et al., 2020; Xu et al., 2023b; Chen et al., 2024; Ingel and Vicente, 2021; Pan et al., 2024; Chuang et al., 2022; Ikeda and Washizawa, 2021; Ding et al., 2021; Wan et al., 2023; Dai et al., 2025), providing a viable alternative to DL approaches. Frequential information within SSVEP signals can be encoded within covariance matrices. As the space of covariance matrices forms a Riemannian manifold (a non-Euclidean metric space), distance-based classification models (such as minimum distance to mean (MDM) algorithms) have been adapted to classify SSVEP using Riemannian distances (Chevallier et al., 2021). Given their combination of simple models and relatively high performance, Riemannian classifiers offer an interesting alternative to their Euclidean counterparts. These SSVEP approaches may be applicable to periodic ERG. However, as they were developed primarily for brain-computer interface, they may not be well suited for medical applications. These signals can also be analyzed using standard classic ML models, using some form of feature extraction (Chen and Golomb, 2023; Scott et al., 2026; Chen et al., 2021; Lei et al., 2024).

### Analysis of multifocal ERG and VEP

**Figure 3:**
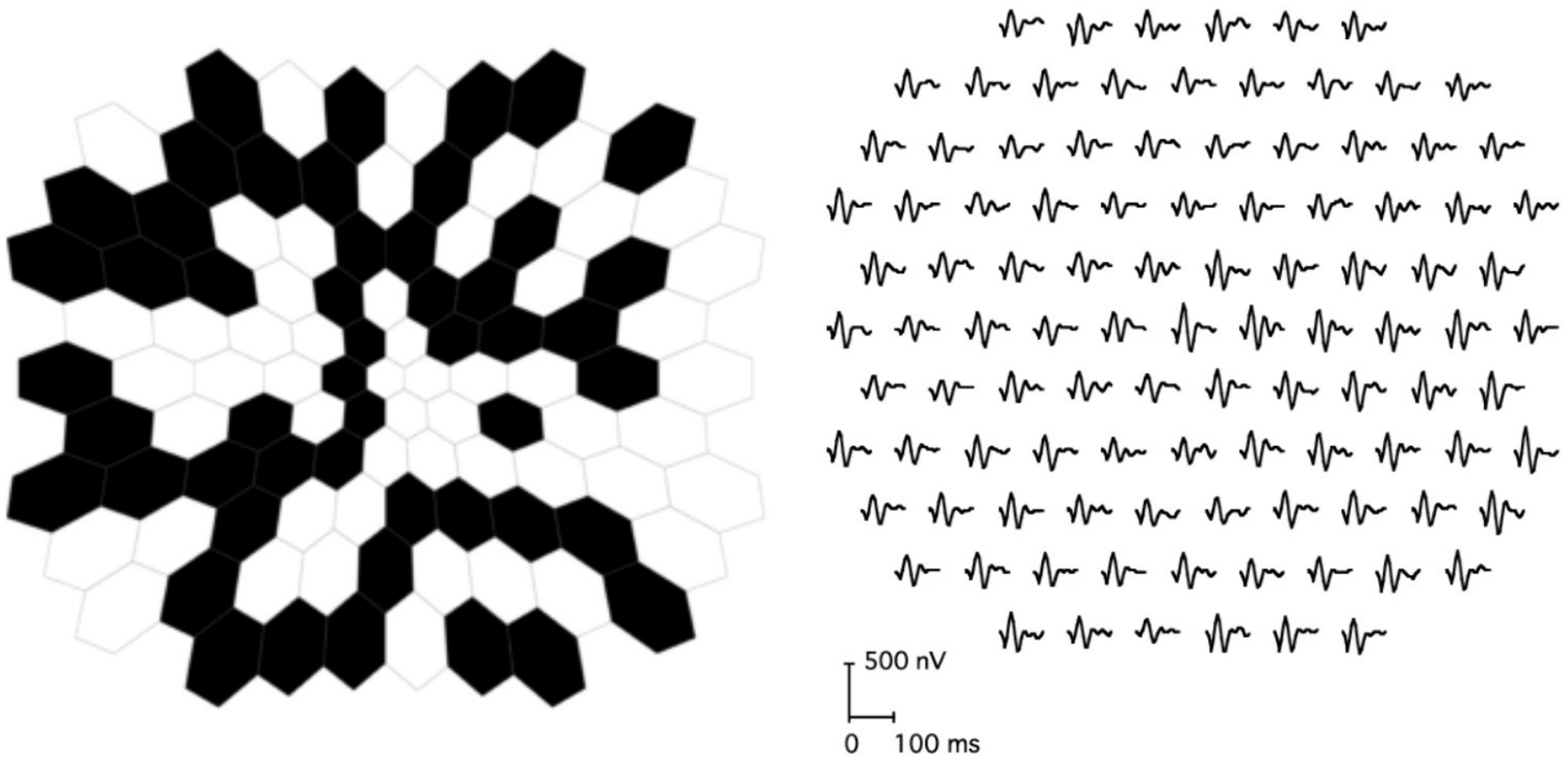
Multifocal ERG stimulus, and isolated responses. Licensed under CC BY 4.0 (Hoffmann et al., 2021), https://creativecommons.org/licenses/by/4.0/, cropped.

Multifocal ERG (mfERG) (Hoffmann et al., 2021) and multifocal VEP (mfVEP) (Hood et al., 2003; Balachandran et al., 2003) utilize an array of black and white cells, yielding a spatial grid of localized transient responses. mfERG probes outer retinal activity (mainly photoreceptors and bipolar cells). It takes the form of a scaled hexagonal grid, with each cell stimulating a different area of the retina (Figure 3). Each cell individually switches intensity following a pseudorandom binary sequence (or m-sequence), such that individual ERG responses for each targeted area may be algorithmically isolated. They serve to analyze localized retinal function by measuring electrical responses from multiple discrete regions of the central retina, primarily the macula. mfVEP assesses cortical responses to localized visual stimuli. It functions similarly to mfERG, though the scaled grid is not necessarily hexagonal. They measure localized cortical responses to visual stimuli across different regions of the visual field, providing a topographic map of visual function. It is used to detect and objectively confirm visual field defects, particularly in cases where standard VEP or perimetry may be inconclusive.

Both mfERG and mfVEP grids can be analyzed through ML approaches adapted to transient signals (Habib et al., 2022; Banijamali et al., 2024; Karaman et al., 2025). Those can be combined with clustering algorithms, to identify patterns within the grid (del Ortiz Castillo et al., 2020). As with multichannel SSVEP, the localized responses can be staked into an image, and fed to image-based models (Klistorner et al., 2022). Furthermore, spatiotemporal and/or graph-based DL models could be used in their analysis.

### Analysis of other types of non-transient ERG and VEP signals

**Figure 4:**
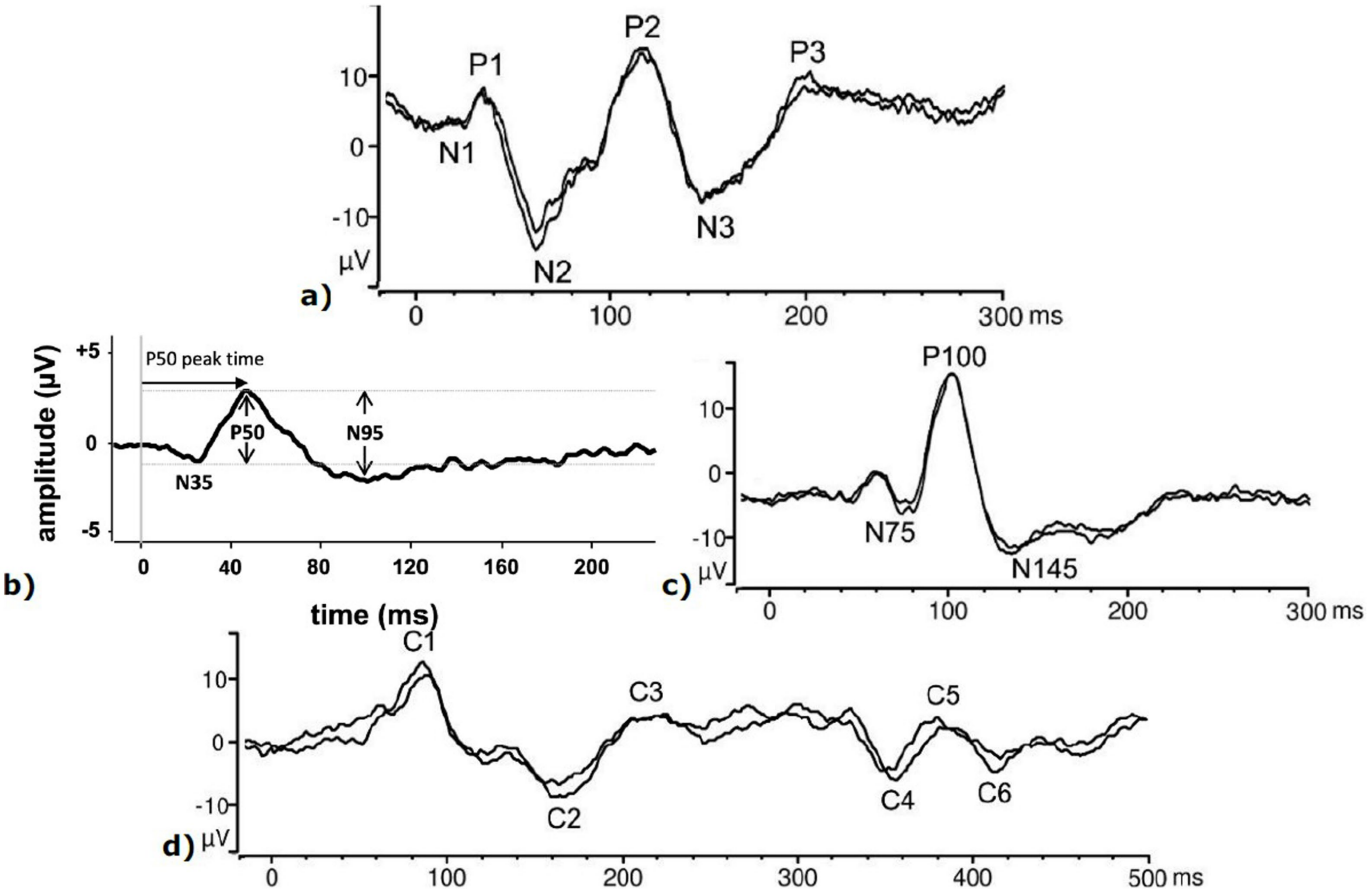
Other transient responses. **(a)** Flash VEP; **(b)** Pattern ERG; **(c)** Pattern VEP; **(d)** pattern onset/offset VEP. Image **(b)** licensed under CC BY 4.0 (Thompson et al., 2024), https://creativecommons.org/licenses/by/4.0/, unchanged. Other images from an accepted ISCEV preprint (Šuštar Habjan et al., 2025).

The remaining VEP subtypes are mostly used for command selection within brain-computer interface. In time-modulated VEP (Bin et al., 2009), several pseudo-random, non-overlapping sequences of flashes are separated enough to induce full FVEP responses. The concentrated-upon stimulus is identified by the sequence of FVEP responses found in the recording (Figure 4). Time-modulated VEP (t-VEP) (Bin et al., 2009) can also be used to investigate the dynamics of visual processing, including temporal sensitivity and selective neuronal adaptation. This approach seems to have fallen out of favor, likely due to its relative slowness compared to alternatives. By contrast, code-modulated VEP (Bin et al., 2009; Martínez-Cagigal et al., 2021) utilize a repeating pseudo-random binary sequence of flashes common to all stimuli, which each stimulus being offset from the others. The concentrated-upon stimulus is detected by identifying the corresponding offset. This approach offers high target differentiation and robustness to interference. Unlike with time-modulated VEP, code-modulated VEP-based brain-computer interface systems can compete and even outperform SSVEP-based systems in both accuracy and speed, but usually require more calibration. Code-modulated VEP analysis can be based on Canonical Correlation Analysis (Martínez-Cagigal et al., 2021; Dong et al., 2025), though other classical ML pipelines (mostly based on support vector machine or linear discriminant analysis) have also been utilized (Martínez-Cagigal et al., 2021); as have DL approaches (Martínez-Cagigal et al., 2021; Dong et al., 2025).

## Conclusion

Each ERG and VEP subtype presents distinct structural properties - transient, periodic, multifocal, or code-modulated - that determine the most appropriate AI approach. Classical machine learning remains valuable for interpretable, feature-based classification, whereas deep learning provides powerful frameworks for modeling complex spatiotemporal dynamics. AI thus enables the extraction of latent patterns, improves the detection of early or focal dysfunction, and enhances reproducibility across operators and centers.

Beyond these methodological advances, several challenges must be addressed to support real-world clinical implementation. Integration into existing electrophysiology workflows requires robust, user-friendly pipelines that interface seamlessly with acquisition systems and reporting tools, while preserving clinician oversight. Regulatory constraints, including data protection, certification of medical AI systems, remain critical considerations. In addition, the limited availability of large, standardized ERG and VEP datasets, reflecting both the niche nature of the field and regulatory barriers to data sharing, continues to hinder model development, external validation, and benchmarking.

Addressing these challenges will be essential to unlock the full clinical potential of AI in visual electrophysiology. With adequately curated datasets, interoperable infrastructures, and clear regulatory pathways, AI could support scalable, sensitive, and personalized assessment of visual pathway function, ultimately enabling reliable deployment in routine practice and large-scale screening settings.
